# Supplementary material for: MetaRibo-Seq measures translation in microbiomes
Source: Nat Commun. 2020 Jun 29;11:3268. doi: 10.1038/s41467-020-17081-z (PMC7324362; doi:10.1038/s41467-020-17081-z)
Supplement: Supplementary file 10 — Supplementary Data 7 [file 41467_2020_17081_MOESM10_ESM.zip › File2/Confidence_High_Taxonomy/201575_out.krona.html]

Javascript must be enabled to view this page.

members
magnitude
magnitudeUnassigned
count
unassigned
taxon
rank

201575\_out

2

superkingdom
2
2

phylum
2
976

200643
class
2

order
2
171549

815
family
2


SRS013687\_contig\_number\_30601
1
2
816
genus

species
1
821

SRS023914\_contig\_number\_19005
